# A Rare Case of Primary Hyperparathyroidism and Hypercalcemia of Malignancy Seen in a Patient With Prostate Adenocarcinoma

**DOI:** 10.7759/cureus.43497

**Published:** 2023-08-14

**Authors:** Asiya Jatoi, Yasser Haider-Badenhorst

**Affiliations:** 1 Endocrinology, Christie Clinic, Urbana-Champaign, USA; 2 Medicine and Surgery, Ziauddin University, Karachi, PAK

**Keywords:** parathyroidectomy, prostate adenocarcinoma (pca), prostatic adenocarcinoma, prostate metastasis, prostate, hyper-parathyroidism, hypo-calcemia, hyper-calcemia, hypo-parathyroidism, parathyroidism

## Abstract

Hypercalcemia of malignancy is commonly associated with several malignancies, but its existence in prostate cancer is an uncommon finding. The concurrent existence of a parathyroid adenoma and a history of hypercalcemia over several decades further adds to the enigma. Our case is of an 82-year-old man with a history of prostate cancer who presented to the endocrinology clinic with hypercalcemia. His PET-CT showed osteolytic metastasis to the T10 vertebrae which were presumed to be the cause of his high serum calcium. Further investigations revealed increased parathyroid hormone-related peptide (PTHrP). Denosumab therapy was started but his calcium remained elevated and hence, he underwent palliative radiation therapy. A follow-up PET-CT revealed significant disease regression and his serum calcium decreased from 11mg/dL to 10mg/dL.

However, one month post radiation his serum calcium started showing an upward trend. Further investigations revealed an elevated parathyroid hormone (PTH) and an ultrasound of the thyroid revealed parathyroid adenoma. The patient subsequently underwent a parathyroidectomy with resolution of hypercalcemia.

## Introduction

Hypercalcemia of malignancy (HCM) is commonly associated with several cancers and its presence has been widely reported in medical literature. Asonitis et al. report the existence of HCM in approximately 20% of all cancers [1]. The etiology can vary, but by far the most common cause is an overproduction of parathyroid hormone-related peptide (PTHrP). Other causes include osteolytic bony metastasis and the production of 1,25-dihydroxy vitamin D, which serves to drive up serum calcium.

Solid tumors most commonly associated with hypercalcemia include squamous cell carcinoma of the head, neck, breast, lungs, ovaries, and renal carcinoma [2]. However, despite its widespread prevalence, the incidence of HCM in prostate cancer has not been well reported and presents as an extremely rare association. A retrospective study conducted using data from the Oncology Services Comprehensive Electronic Records (OSCER) warehouse of electronic health records established that prostate cancer had the lowest rates of HCM at 1.4-2.1% [3].

Moreover, hypercalcemia associated with prostate cancer is most often attributed to an elevated PTHrP, as the bony metastasis associated with the tumor is osteoblastic in nature. It is extremely rare to see prostate cancer presenting with osteolytic metastasis as the cause of hypercalcemia [4].

Here, we present a case of hypercalcemia of malignancy associated with osteolytic prostate carcinoma and the concurrent existence of primary hyperparathyroidism.

## Case presentation

An 82-year-old man presented to the endocrinology clinic in May 2023 for a workup of hypercalcemia. His associated symptoms included constipation, increased thirst, polyurea, poor concentration, nausea, and fatigue. Examination revealed a hypertensive male (153/86 mmHg) with a BMI of 28.31kg/m2. Review of systems was unremarkable, and his history did not reveal prescription of any drugs known to cause hypercalcemia.

**Figure 1:**
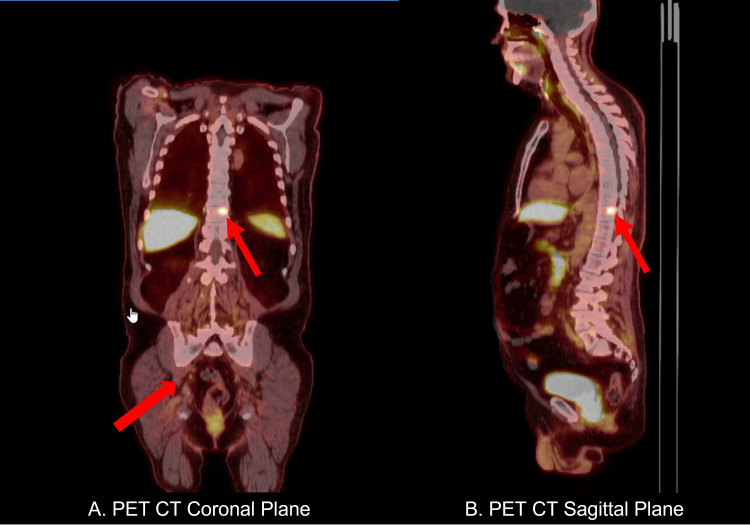
PET-CT showing metastatic osteolytic lesions in the left anterior pubic ramus and T10 vertebrae

He had a history of Gleason grade 4B prostatic adenocarcinoma which was diagnosed in 2021. A PET-CT (Figure 1) performed in April 2022 revealed metastatic osteolytic lesions in the left anterior pubic ramus and T10 vertebrae, and he was subsequently started on enzalutamide therapy. His basic metabolic panel revealed an elevated serum calcium (11.5mg/dL), which was treated with monthly denosumab 120mg and a single dose of IV pamidronate 90mg. Although not a common association, a PTHrP level was sent which was elevated at 22 pg/mL (range 11-20 pg/mL) and alkaline phosphatase was 117U/L (range 25-100 U/L). He was thus diagnosed with humoral hypercalcemia of malignancy (HHM). Although medical therapy initially showed a decrease in serum calcium levels, a few months later, the patient remained symptomatic and started showing increasing serum calcium levels.

The patient received palliative radiation therapy to the T9-11 vertebra in July 2022, and a total dose of 3000 cGy was provided in 10 fractions. Post radiation therapy, his serum calcium decreased from 11mmol/L to 10mmol/L, which further confirmed the diagnosis of hypercalcemia of malignancy.

However, soon after, his calcium (11mg/dL) and parathyroid hormone (PTH) (108pg/dL) were both elevated. Serum alkaline phosphatase and albumin remained within normal limits. A detailed personal history and review of medical records led to the discovery of a mildly elevated serum calcium (10.5mg/dL) since 2001 leading to the belief that subclinical hyperparathyroidism had been present for over two decades but was only brought to medical attention when serum calcium levels reached clinical significance in 2021, owing to hypercalcemia of malignancy. Further evaluation revealed prostate cancer in both his brothers and breast cancer in his daughter who had tested positive for a mutation in the HOXb9 gene. The HOXb9 gene encodes a nuclear protein that functions as a sequence-specific transcription factor and is involved in cell proliferation and differentiation. Increased expression of the gene is associated with an increased incidence of breast and prostate cancers [5]. The patient did not undergo genetic testing, but the strong family history suggests the presence of the mutation.

In light of his long-standing history of hypercalcemia, a diagnosis of familial hypocalciuric hypercalcemia (FHH) was considered. The condition is caused by defective G-coupled calcium-sensing receptors in multiple tissues including the parathyroid glands and the kidneys. This was an important consideration as the presence of FHH is an absolute contraindication to performing a parathyroidectomy [6], because the procedure provides no therapeutic benefit. The hallmark of the disease is an elevated PTH and serum calcium, and low urine calcium. A 24-hour urine calcium level was thus assessed and revealed an elevated level at 304mg/24hrs, effectively ruling out the condition. 

**Figure 2:**
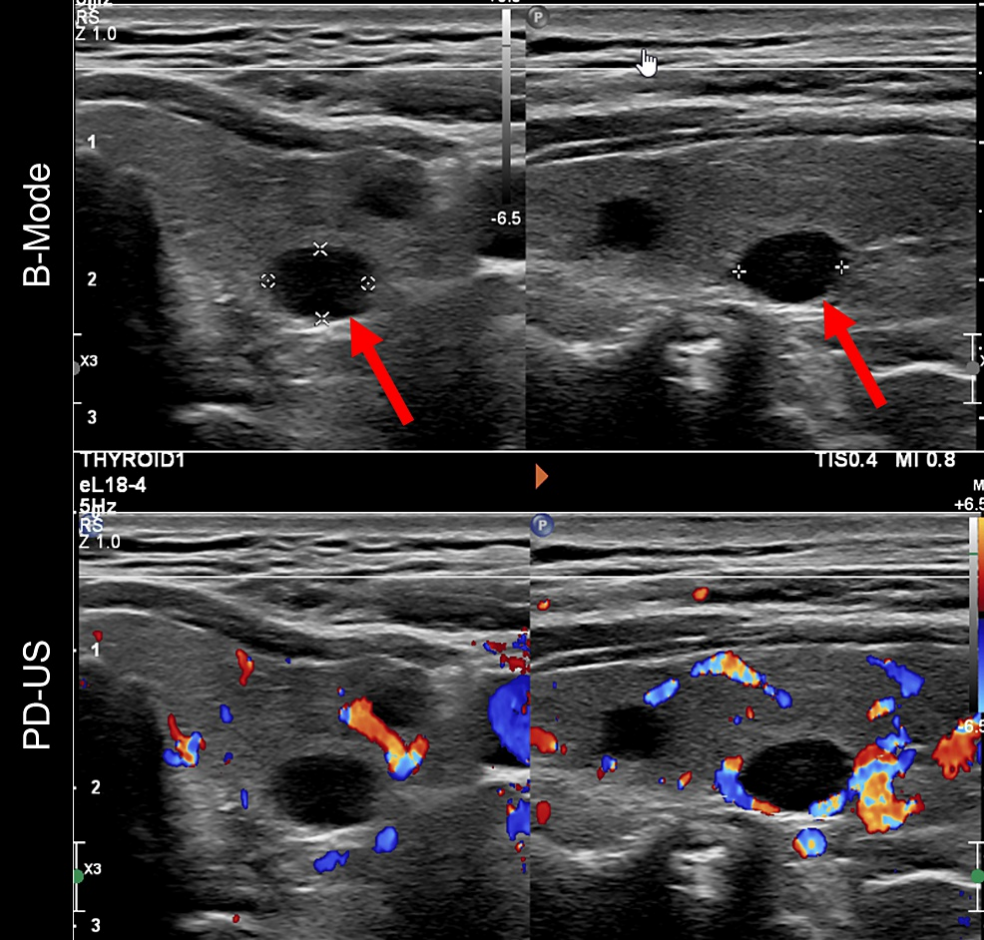
Ultrasound of the thyroid showing a 0.8 cm hypervascular solid nodule in the left lower pole

**Figure 3:**
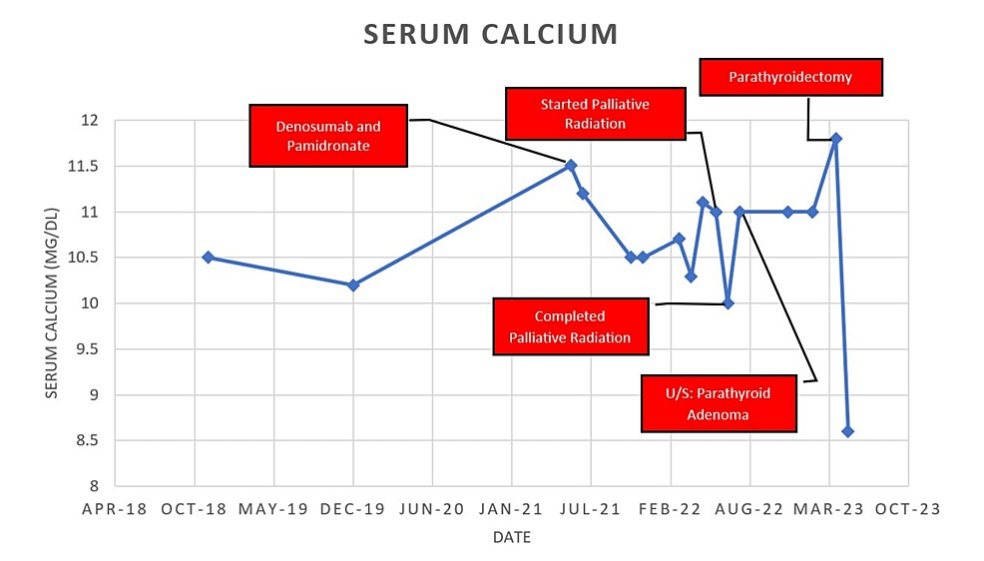
Serum calcium trends over the length of treatment.

A preliminary diagnosis of primary hyperparathyroidism and concurrent hypercalcemia of malignancy was made. An ultrasound of the thyroid was conducted, shown in Figure 2, which revealed a 0.8 cm hypervascular solid nodule possibly representing parathyroid adenoma in the left lower pole. The patient underwent a parathyroidectomy in April 2023. His intraoperative PTH was 107pg/dL, and postoperative readings showed a drastic decrease to 8pg/dL. The procedure led to a resolution of the patient’s symptoms and normalization of serum calcium (8.6mg/dL). Histopathology later confirmed the presence of a parathyroid adenoma. Mild hypocalcemia postoperatively was managed with Tums (calcium carbonate) and calcitriol, which were soon discontinued. Figure 3 shows the serum calcium trends over the course of the patient's treatment.

## Discussion

Prostate cancer is the most common cancer in men worldwide and the second most common cause of cancer-associated mortality in the same demographic [7]. These statistics highlight the importance of better understanding the varied presentations of the disease. Although prostate cancer has a propensity to cause osteoblastic lesions, osteolytic lesions are also a possible manifestation. The existence of osteolytic metastasis and the secretion of PTHrP are the two proposed mechanisms leading to hypercalcemia.

Hypercalcemia of malignancy is associated with a poor prognosis. A retrospective study conducted on 126 patients treated for hypercalcemia of malignancy revealed a median survival of 30 days [8]. Palliative anti-hypercalcemic drugs and radiation can decrease the tumor burden and lead to symptomatic improvements. However, the concurrent existence of a parathyroid adenoma can present as a therapeutic challenge and cause errors in appropriate diagnosis. It is therefore critical to differentiate between the two.

Parathyroid adenomas function by secreting excess PTH, which serves to raise serum calcium by increasing its absorption from the gastrointestinal tract, kidneys, and bones. Management depends on the patient’s symptomatic presentation as well as the level of hypercalcemia. Our patient presented to the endocrinology clinic with symptomatic hypercalcemia which was attributed to his cancer. The association between his serum abnormalities and cancer was established by an elevated PTHrP, the presence of osteolytic metastasis and a significant decrease in serum calcium following radiation therapy. Although therapy aimed at reducing tumor burden did cause an initial decrease, serial monitoring showed an increase in calcium after a month. A subsequent parathyroidectomy led to the resolution of his symptoms.

This case is a rare example of the concurrent existence of two primary causes of hypercalcemia in the same patient. It is important to approach hypercalcemia with consideration of all possible causes, so treatment may be catered accordingly.

## Conclusions

Due to the high variability in prostate cancer presentation, a high index of suspicion should be maintained when a patient presents with hypercalcemia. Although several cancers have been commonly linked to osteolytic bony metastasis and hypercalcemia, prostate cancer is a rare association that should not be overlooked.

Furthermore, the concurrent existence of two primary causes of hypercalcemia such as a parathyroid adenoma and malignancy can present as a diagnostic challenge that needs further evaluation. Management of both causes independently can lead to symptomatic improvement.
